# Determination of Aflatoxin B1 Levels in Organic Spices and Herbs

**DOI:** 10.1155/2013/874093

**Published:** 2013-05-26

**Authors:** Halil Tosun, Recep Arslan

**Affiliations:** ^1^Department of Food Engineering, Faculty of Engineering, Celal Bayar University, Manisa 45140, Turkey; ^2^Graduate School of Natural and Applied Science, Celal Bayar University, Manisa 45140, Turkey

## Abstract

Organically produced spices and herbs were analyzed for determination of aflatoxin B1 (AFB1) by ELISA using immunoaffinity column. For this purpose 93 organic spices and 37 organic herbs were randomly selected from organic markets and organic shops in Turkey. AFB1 was detected in 58 organic spice and 32 organic herb samples. Among organic spice samples, the maximum value was detected in cinnamon sample (53 **μ**g/kg). AFB1 was not detected in thyme samples. AFB1 levels of 41 organic spice samples were above the EU regulatory limit (5 **μ**g/kg). Among organic herb samples the highest concentration of AFB1 (52.5 **μ**g/kg) was detected in a rosehip sample. AFB1 levels of 21 organic herb samples were above the regulatory limits of the European Union. These results showed that more stringent measures must be taken for the prevention of mold contamination in the production of organic spices and herbs.

## 1. Introduction

Because of their preharvest, postharvest, and storage conditions, spices and herbs can be contaminated with mycotoxins. It has been reported that 5–10% of agricultural products in the world are contaminated by moulds to the extent that these products cannot be consumed by humans and animals [1]. Spices are largely produced in countries with tropical climates that have high range of temperature, humidity, and rainfall [2]. Furthermore, improper storage, extended drying times, and elevated moisture contents may cause development of mycotoxins in spices and herbs. Aflatoxin is the most common mycotoxins in spices, and aflatoxin contamination in spices has been studied by several researches [3–5]. Aflatoxins are mycotoxins produced by the fungi *Aspergillus flavus*, *Aspergillus parasiticus*, and *Aspergillus nomius. *These mycotoxins are teratogenic, mutagenic, and carcinogenic in humans and animals [6, 7]. The most toxic and carcinogenic member of this family, aflatoxin B1, is acutely poisonous, highly mutagenic, and intensely carcinogenic in rodents and other animals and is classified by the International Agency for Research on Cancer in the group of molecules that are carcinogenic for both human, and animals [8, 9].

Organic foods are grown without synthetic antifungals [10]. In fact organic crops are claimed to be more vulnerable to mold contamination because synthetic fungicides cannot be used in their production.

The objective of this study was to investigate AFB1 contamination in organic spices and herbs.

## 2. Materials and Methods

### 2.1. Sample Collection

Between May 2010 and May 2011, 93 organic spice and 37 organic herb samples were randomly obtained from organic product markets and organic shops from Turkey. All the samples have organic food label. Samples were stored at 4°C until further analysis.

### 2.2. Analysis of AFB1 by ELISA

Preparation of the samples and separation with immunoaffinity column procedure were performed according to the method described by R-Biopharm [11].

### 2.3. Evaluation of Data

AFB1 levels of samples were evaluated according to the Gen5 computer program prepared by Biotek Instruments, Inc. The detection limit of the kit for spices and herbs was 0.025 *μ*g/kg and recovery rate was 70–110%.

### 2.4. Preparation of Samples

5 g of ground sample was added to 25 mL methanol (70%), and the solution was extracted by shaking for 10 min. The extract was filtered through a fluted paper filter, and 15 mL distilled water was added to 5 mL of the filtered solution. Afterwards, 0.25 mL Tween 20 was added to the filtered solution, and it was stirred for 2 min.

### 2.5. Separation with Immunoaffinity Column

The immunoaffinity column was rinsed with 2 mL distilled water, and then the column was filled with approximately 1 mL prepared sample solution. Suitable adapter was attached on top of the column, and syringe was used as sample reservoir. Then, syringe was filled with the residual sample solution. Sample extract was passed slowly and continuously through the column (flow rate: approximately 1 drop/sec). The column was rinsed with 10 mL distilled water, and the passed solution was discarded. The column was dried by passing air through the column for 10 s. The syringe was removed, and a clean and closable vial directly was placed below the column. 0.5 mL of methanol was passed slowly through the column (flow rate: approximately 1 drop/sec). Toxin containing eluate was diluted tenfold with distilled water (50 *μ*L + 450 *μ*L distilled water) and used 50 *μ*L per well in the assay.

### 2.6. Test Procedure

According to Ridascreen Aflatoxin B1 (Art No: 1211) test kit manual, 50 *μ*L standard or prepared samples was pipetted into separate wells in duplicate. Then, 50 *μ*L of enzyme conjugate and 50 *μ*L of anti-aflatoxin antibody solution were added to each well, mixed gently, and incubated for 30 min at 25°C in the dark. The liquid was poured out of the wells, and then wells were washed twice with 250 *μ*L washing buffer. 100 *μ*L substrate/chromogen was added to each well. Plate was incubated for 15 min at 25°C in the dark. Finally 100 *μ*L of stop solution was added to each well, and the absorbance was measured at 450 nm (ELx 800, BioTek Instruments, USA).

## 3. Results and Discussion

The AFB1 content of the organic spices and organic herbs is summarized in Tables 1 and 2. AFB1 was detected in 58 of 93 organic spice samples (62%) and 32 of 37 organic herb samples (86%). AFB1 levels in 41 (44%) of 93 organic spice samples and 21 (57%) of 37 organic herb samples were above the regulatory limit, which had been set at 5 *μ*g/kg for AFB1 in European Commission [12]. AFB1 was detected in all the basil, cinnamon, camomile, and sage samples. AFB1 was not detected in any of the thyme samples analyzed. In organic spices, cinnamon had the highest mean concentration of AFB1 (51.6 *μ*g/kg) while in organic herbs, rosehip had the highest mean concentration of AFB1 (44.5 *μ*g/kg).

The popularity of organic foods continues to grow dramatically. Consumers purchasing organic foods may do so for a number of reasons, including perceived benefits to the environment, animal welfare, and worker safety and the perception that organic foods are safer and more nutritious [10]. This perception is mainly associated with organic food production techniques. In organic food production the use of synthetic fertilizers or sewage sludge is prohibited [13]. Organic foods, despite the assumption that they are safe, can also carry risks as much as conventional foods. Some studies have concluded that organic foods are significantly more contaminated with mycotoxins than conventional foods [14–19].

In our study the results of the survey indicate that organically produced spices and herbs were heavily contaminated with AFB1, especially cinnamon, sumac, red pepper, camomile, and rosehip samples. Several studies have reported AFB1 contamination in spices and herbs. Ozbey and Kabak [5] reported that AFB1 levels in four red chili flake and three red chili powder samples were above the EU limit of 5 *μ*g/kg. Aydin et al. [20] analyzed 100 powdered red pepper samples in Turkey, and AFB1 levels in 18 (18%) of 100 powdered red pepper samples were found to be higher than the legal limits of European Commission (>5 *μ*g/kg). They reported high levels of AFB1 contamination in red pepper powder with levels of contamination up to 40.9 *μ*g/kg. Kanbur et al. [21] reported that the AFB1 contamination in red pepper samples is from 1.48 to 70 *μ*g/kg in Turkey. Erdogan [22] reported that 8 out of 44 red scale pepper samples were contaminated with AFB1 ranging from 1.1 to 97.5 *μ*g/kg. Riordan and Wilkinson [23] tested 130 commercial spice preparations, and 96% of the samples contained aflatoxin <10 *μ*g/kg. Maximum aflatoxin level was detected (27.5 *μ*g/kg) in chili powder. Martins et al. [2] reported that cumin samples were contaminated with AFB1 in the range of 1–5 *μ*g/kg in Portugal. In our study, among samples, poppy seeds, anise, and thyme had the lowest AFB1 value. The absence of AFB1 contamination in thyme samples may be attributed to inhibition of mold growth by indigenous antifungal activity of essential oils. R. Z. Soad and M. A. Soad [24] reported that thyme oil was effective against all the tested fungi, and thyme oil could be used as a suitable lead to design effective and specific new fungicides. Anise essential oil has also stronger antifungal activities as several studies have shown [25–27]. 

## 4. Conclusion

The mycotoxin risks of organically produced foods are real due to the increasing popularity of organic food. Organic farming methods can potentially lead to fungal contamination because synthetic fungicides are not allowed in organic production. Our study showed that organic spices and herbs could be contaminated by AFB1. Occurrence of AFB1 in organic spices and herbs indicated the importance of good organic agricultural practice including using new effective antifungal agents. 

## Figures and Tables

**Table 1:** Aflatoxin B1 content of organic spice samples.

Organic spices	*n*	Positive samples *n* (%)	No. of samples above EU limit n (%)	Range of AFB1 concentration in positive samples (*µ*g/kg)	Mean of positive samples (*µ*g/kg)
Laurel leaves	7	2 (29)	2 (29)	16.5–20.3	18.4
Cumin	8	5 (62)	2 (25)	0.5–26.3	10.7
Mint	5	4 (80)	2 (40)	4.2–26.7	14.7
Rosemary	6	3 (50)	2 (33)	3.3–10	6.7
Basil	6	6 (100)	2 (33)	0.8–18.1	7.5
Cinnamon	5	5 (100)	5 (100)	49.4–53	51.6
Poppy seeds	7	3 (43)	0 (0)	0.98–3.2	2.4
Thyme	6	0 (0)	0 (0)	ND	ND
Ginger	4	3 (75)	2 (50)	3.8–23.1	16.5
Anise	6	4 (67)	3 (50)	4.9–8.4	7.1
Sumac	10	8 (80)	7 (70)	51.2–52.5	45.8
Black pepper	6	4 (67)	4 (67)	24.6–30	27.6
Red pepper flakes	7	4 (57)	3 (43)	3.5–30.3	23.4
Red pepper	8	6 (75)	6 (75)	23.4–46.6	41.5
Coriander	2	1 (50)	1 (50)	15.6–15.6	15.6

Total	93	58 (62)	41 (44)		

ND: not detected.

**Table 2:** Aflatoxin B1 content of organic herb samples.

Organic herbs	*n*	Positive samples *n* (%)	No. of samples above EU limit *n* (%)	Range of AFB1 concentration in positive samples (*µ*g/ kg)	Mean of positive samples (*µ*g/kg)
Linden	5	3 (60)	1 (20)	0.05–40.6	14
Fennel seeds	7	6 (86)	3 (43)	1.1–11	5.7
Camomile	10	10 (100)	9 (90)	3.4–38.9	28.7
Sage	9	9 (100)	4 (44)	0.2–32.2	8.9
Rosehip	6	4 (67)	4 (67)	20.7–52.5	44.5

Total	37	32 (86)	21 (57)		
